# P-1004. The Ascendance of Serotype VIII in Group B Streptococcus among Korean Women: A Detailed Examination of Serotype Distribution and Multilocus Sequence Typing

**DOI:** 10.1093/ofid/ofae631.1194

**Published:** 2025-01-29

**Authors:** Byung Ok Kwak, Dong Hyun Kim

**Affiliations:** Kangnam Sacred Heart Hospital, Seoul, Seoul-t'ukpyolsi, Republic of Korea; Inha University School of Medicine, Incheon, Inch'on-jikhalsi, Republic of Korea

## Abstract

**Background:**

Group B Streptococcus (GBS) remains a formidable agent in neonatal sepsis and meningitis, often transmitted from mothers to neonates. Despite traditionally lower colonization rates in pregnant Korean women compared to their Western peers, emerging evidence suggests an uptick in both colonization rates and serotype variability. This study endeavors to elucidate the patterns of serotype distribution and sequence types among both pregnant and non-pregnant Korean women.

**Methods:**

We analyzed 115 specimens, comprising 72 vaginal and 43 anorectal swabs, collected from 85 pregnant and 10 non-pregnant women. Serotyping was executed via multiplex PCR, complemented by multilocus sequence typing (MLST) of the GBS isolates.

**Results:**

In our cohort of pregnant women, the leading serotypes were VIII (24.7%), followed by V (21.2%), III (20%), Ib (12.9%), and Ia (10.6%). Lesser prevalent serotypes included II (4.7%), IV (2.4%), and VI (2.4%). The sequence types most frequently encountered were ST2 (22.4%) and ST19 (14.1%), with ST2 predominantly associated with serotype VIII, and ST19 with serotypes III and V. Among non-pregnant women, the distribution was serotype VIII and Ia (20% each), followed by Ib (20%), III, IV, and V (10% each).

**Conclusion:**

Contrary to earlier observations, serotype VIII has now emerged as the primary serotype among Korean women, succeeded by serotypes V, III, Ib, and Ia. Our findings underscore the imperative for continued and extensive surveillance to refine our understanding of the epidemiological and serological dynamics of GBS within this population.

**Disclosures:**

**All Authors**: No reported disclosures

